# The level of dental fear and anxiety is higher in children with both severe Molar-Incisor Hypomineralisation and active dental caries lesions compared to children without these conditions

**DOI:** 10.1007/s40368-024-00923-5

**Published:** 2024-08-29

**Authors:** Ó. A. Rodríguez, M. Laverde, D. F. Rojas-Gualdrón, J. M. Cárdenas, J. D. Mejía, A. L. de Farias, L. Santos-Pinto, M. Restrepo

**Affiliations:** 1 Basic and Clinical Research Group in Dentistry, School of Dentistry, CES University, Medellín, Colombia; 2 School of Medicine, CES University, Medellín, Colombia; 3 Department of Morphology, Genetics, Orthodontics and Pediatric Dentistry, São Paulo State University (Unesp), Araraquara School of Dentistry, Araraquara, São Paulo Brazil; 4 Facultad de Odontología, Universidad CES, Calle 10A #22-04, Medellín, Colombia

**Keywords:** Behaviour, Child, Dental anxiety, Developmental defects of enamel

## Abstract

**Purpose:**

To assess levels of dental fear and anxiety (DFA) in children with and without Molar-Incisor Hypomineralisation (MIH) and dental caries lesions.

**Methods:**

In this cross-sectional observational study, 159 children between 8 and 12 years of age were included. For the evaluation of DFA, children responded to the validated version of the Children’s Fear Survey Schedule-Dental Subscale. MIH was assessed using the MIH Index. To evaluate the activity of dental caries lesions and dental caries experience, the Nyvad criterion and the dmft/DMFT index were used, respectively. Dental hypersensitivity was evaluated using air stimulation and a Visual Analogue Scale. The association between MIH and dental caries with DFA was assessed using the generalised linear model with Poisson family, identity link function and robust variance estimation. The significance level was set at 5%.

**Results:**

The mean DFA score was 28.3 (SD = 13.4) with scores ranging from 15 to 64. Amongst children presenting both MIH and dental caries, the perception of DFA was notably higher compared to those with either MIH or dental caries alone. The activity of caries lesion in patients with MIH also influenced DFA levels (diff: 18.6; 95% CI: 12.0–25.2; *p* < 0.001). Dental caries experience in the primary dentition also demonstrated statistical significance concerning DFA (95% CI: 0.8–13.3; *p *value = 0.027).

**Conclusion:**

Children with MIH exhibit higher levels of DFA than children without MIH. The experience of dental caries and the activity of caries lesions significantly influence the perception of DFA in children with MIH.

## Introduction

Dental fear describes a reaction to a stimulus perceived as threatening, and dental anxiety refers to a state of apprehension that occurs before a dental visit or treatment (Klingberg and Broberg 2007). In children, it is challenging to distinguish between these two states, likely because they experience different combinations of fear and anxiety responses. For this reason, the term ‘dental fear and anxiety’ (DFA) has been suggested to describe the negative feelings associated with dental treatment (Klingberg and Broberg 2007). It is estimated that approximately 9% of children and adolescents exhibit DFA (Klingberg and Broberg 2007).

The aetiology of DFA is multifactorial, influenced by exogenous factors, including direct learning experiences (e.g. traumatic experiences) and indirect learning (e.g. vicarious learning), as well as endogenous factors such as genetics, personality traits, age and gender (Campbell 2017). The child’s thoughts and behaviours, as well as the patient–dentist relationship, have also been shown to play a significant role in maintaining DFA over time (Campbell 2017).

DFA has implications for patients, families, dentists and healthcare services (Campbell 2017). Children with DFA often face behavioural management issues, resulting in delays or even barriers to dental treatment. Consequently, the patient’s oral health situation can worsen, requiring more complex and costly treatments. In addition, it can affect oral health-related quality of life (Campbell 2017).

Molar-Incisor Hypomineralisation (MIH) is a multifactorial developmental enamel defect that affects at least one permanent first molar and frequently, permanent incisors (Weerheijm et al. 2001; Bussaneli et al. 2022). Clinically, it is characterised by the presence of demarcated opacities, and in severe cases, it is associated with post-eruptive breakdown, dental caries and hypersensitivity (Weerheijm et al. 2001; Bussaneli et al. 2022; Lygidakis et al. 2022). Due to alterations in the structure and composition of hypomineralised teeth, patients affected by MIH present a higher risk of dental caries than patients without MIH (Villani et al. 2023). In addition, it has been demonstrated that patients affected by severe MIH often encounter dental behaviour management problems and require increased treatment (Bussaneli et al., 2022; Reis et al. 2023; Jälevik et al. 2022; Jälevik and Klingberg, 2002). Therefore, the study of DFA in children and adolescents, particularly in patients with severe MIH, is pertinent given the complexity of this type of enamel developmental defect and its association with dental caries (Bussaneli et al., 2022; Villani et al., 2023).

Although two systematic reviews showed no difference in DFA levels between patients with and without MIH, attention should be paid to the assessment of evidence certainty, which has been reported as *very low* (Reis et al. 2023; Jälevik et al. 2022). Reis et al. highlighted that published studies have limitations regarding sample size, risk of bias, comparability and diagnostic criteria for MIH (Reis et al. 2023). The authors also note that, whilst the results represent the current state of evidence, they are not yet conclusive (Reis et al. 2023).

Understanding and addressing DFA in children affected by MIH are essential to provide a dental experience within an empathetic and patient-centred clinical practice. In this context, the objective of this study was to assess levels of DFA in children with and without Molar-Incisor Hypomineralisation (MIH) and dental caries lesions.

## Materials and methods

This study is reported following the STROBE (Strengthening the Reporting of Observational Studies in Epidemiology) guidelines.

### Study design and setting

This cross-sectional observational study was conducted at the dental clinic of the Department of Pediatric Dentistry at the School of Dentistry of XXX University (City, Country). The research was approved by the University’s Human Ethics Committee (Minute #109; project code: 818). Parents provided informed consent before participating in the study. Data collection began in September 2019, was interrupted in March 2020 due to the coronavirus pandemic, resumed in January 2021, and concluded in April 2023, totalling 36 months.

### Participants

Children aged between 8 and 12 years old with all four first permanent molars fully erupted were included. Children with fixed orthodontic or orthopaedic appliances, diagnoses of syndromes, genetic disorders, neurological disorders, psychological disorders or diseases were not included. Children with poorly completed or incomplete clinical records were excluded.

### Variables

The primary outcome was the perception of DFA. Confounding variables considered included the severity of MIH, dental caries (activity and experience in both primary and permanent dentition), dental hypersensitivity, age, sex and parents’ level of education.

### Data source/measures

To assess sociodemographic characteristics, the parents completed a form in which they recorded the child’s sex, age, level of education, socioeconomic status, family residential area, parents’ level of education, age and marital status.

For the assessment of DFA, children responded to the Children’s Fear Survey Schedule-Dental Subscale (CFSS-DS) (Cuthbert and Melamed. 1982) validated for Colombian Spanish (Cárdenas-Vásquez et al. 2009). This instrument includes 15 items related to dental treatment, with responses scored on a scale from 1 (no fear) to 5 (much fear). The total score can range from 15 to 75 points (Klingberg and Broberg. 2007; Reis et al. 2023).

MIH was assessed and classified according to the long version of the MIH Index, following the clinical examination protocol described by Ghanim et al. (Ghanim et al. 2015, 2017). This index is validated and considers the eruption status, clinical state and surface extent of the defect (Ghanim et al. 2019). In addition, it distinguishes MIH from diffuse opacities (dental fluorosis), hypoplasia, amelogenesis imperfecta and other types of hypomineralisation (Ghanim et al. 2015). Severity classification was done according to Ghanim et al. (Ghanim et al. 2017). Children with only teeth displaying colour changes were classified as mild, whilst those with at least one tooth with structural loss and/or atypical carious lesions or extractions were classified as severe (Ghanim et al. 2017).

The dental caries lesions were evaluated and classified according to the Nyvad criteria (Nyvad et al. 1999). This criterion is validated and involves visual and tactile assessment, considering both the activity and severity of caries lesions. The classification is based on the following codes: 0 = sound; 1 = active caries (intact surface); 2 = active caries (surface discontinuity); 3 = active caries (cavity); 4 = inactive caries (intact surface); 5 = inactive caries (surface discontinuity); 6 = inactive caries (cavity); 7 = filling (sound surface); 8 = filling + active caries and 9 = filling + inactive caries (Nyvad et al. 1999; Nyvad et al., 2018).

Dental caries experience was assessed using the decayed, missing and filled teeth in primary dentition (dmft) and decayed, missing and filled teeth in permanent dentition (DMFT) index proposed by the World Health Organisation (World Health Organization. 2013). The clinical examination was performed by the same examiner (O.R) and assistant who assessed MIH.

To evaluate dental hypersensitivity, the examiner (O.R) applied a blast of room-temperature air (19 − 24 °C) for 1 s at a distance of 1 cm, perpendicular to the tooth surface (Bekes et al. 2017). Immediately after, the child subjectively rated the pain on a Visual Analogue Scale (VAS). This scale consists of a horizontal line of 10 cm with two points, one at the beginning and the other at the end, representing “no pain” and “worst pain,” respectively. To obtain the final classification, the distance in centimetres between the starting point and the mark DFA by the child was measured (Raposo et al. 2019; de Farias et al. 2021).

### Biases

The assessment and classification of MIH were carried out by an examiner with clinical experience in paediatric dentistry and in conducting clinical and epidemiological studies on enamel development defects. The examiner (O.R) was calibrated by an expert researcher (A.L.F) in the use of the MIH Index, following the recommendations of the specific training manual for this index previously published by Ghanim et al. (Ghanim et al. 2015). Calibration involved the use of 115 clinical photographs of sound permanent teeth and those with various types of enamel development defects. The examiner (O.R) evaluated the photographs twice with a 1-week interval after the first assessment and achieved an intra-examiner Kappa value of 0.87 for both clinical presentation and defect extent. The inter-examiner Kappa value (examiner vs. expert researcher—A.L.F) was 0.85 and 0.81 for clinical presentation and defect extent, respectively.

The same examiner (O.R) was calibrated by another expert researcher for the assessment and classification of dental caries lesions using the Nyvad criteria (Nyvad et al. 1999). For this calibration, 78 previously validated clinical photographs were used and were evaluated twice with a 1-week interval after the first assessment. The intra-examiner Kappa value was 0.93, and the inter-examiner Kappa value (vs. the expert researcher) was 0.90. Following the same methodology, the examiner was calibrated for the use of dmft/DMFT index, obtaining an intra-examiner and inter-examiner Kappa of 0.98 and 0.97, respectively.

Once the examiner’s calibration (O.R) was completed, a pilot study was conducted with ten children of the same age and from the same population as those in the main study to assess the applicability of the instrument to evaluate MIH and the criteria to evaluate and classify MIH, dental caries and dental sensitivity. The results of the pilot study did not suggest changes in the study methodology.

### Study size

A priori sample size estimation was not performed as all eligible patients from the dental clinic were included. Post hoc analysis established a statistical power greater than 0.80 for absolute mean differences ≥ 3.5 points on the MIH scale.

### Statistical analysis

Descriptive analysis was conducted using the median and interquartile range (IQR) for quantitative variables, and frequencies and percentages for categorical variables. The prevalence of MIH and dental caries (dmft/DMFT) was estimated at the child level (child with at least one affected tooth). In addition, the average dmft, DMFT and dental sensitivity are presented. For dental characterisation, MIH was classified as healthy, mild and severe (Ghanim et al. 2017). Dental caries lesions were classified as healthy, active and inactive according to the Nyvad criteria (Nyvad et al. 1999).

The association between MIH and dental caries with DFA was analysed using a generalised linear model with a Poisson family, identity link function, and robust estimation of variance since DFA did not follow a normal distribution (Shapiro–Wilks *p *value < 0.001). Mean differences with 95% confidence intervals (95% CI) are presented for observed and adjusted estimates. Covariates showing a *p *value < 0.25 were included in the adjusted model. In addition, the interaction between MIH and dental caries with respect to DFA was explored in the adjusted regression model.

The significance level was set at 0.05. The analyses were performed using Stata version 16.1.

## Results

### Participants

Out of a total of 965 children, 274 initially met the eligibility criteria outlined by the study. Amongst them, 53 were excluded due to the presence of fixed orthodontic or orthopaedic appliances, whilst 44 were excluded for exhibiting enamel developmental defects other than MIH. Furthermore, 12 individuals were excluded due to special needs, and 6 were excluded due to inconsistencies in the sociodemographic information provided by their parents. As a result, the final sample consisted of 159 children.

### Descriptive data

Table 1 presents the sociodemographic characteristics of the children. Of the total, 59.1% were boys; the median age was 9 years (IQR 8–11), and 68.6% were in primary education. Table 2 presents the characteristics regarding MIH and dental caries. 76% of the children were free of MIH, 19.5% had at least one tooth with severe MIH, and 3.8% had at least one tooth with mild MIH. The prevalence of dental caries was 29.6% with average values of 0.3 (SD = 0.9) and 4.4 (SD = 16.8) for DMFT and dmft, respectively. The prevalence of active and inactive dental caries lesions was 8.2% and 1.3%, respectively. When analysing the combined presentation, it was found that 7.5% of the patients presented severe MIH and active dental caries. Regarding dental hypersensitivity, the mean was 1.1 (SD = 2.8).

**Table 1:** Sociodemographic characteristics

	n	%
Gender		
Female	65	40.9
Male	94	59.1
Age		
Median, IQR	9	(8–11)
Socioeconomic level		
Low	34	21.4
Middle	95	59.8
High	30	18.9
Residential area		
Rural	27	17.0
Urban	132	83.0
Child´s education		
Primary	109	68.6
High school	50	31.5
Father´s education		
Elementary	15	9.4
Graduate	42	26.4
Technical or technological	26	16.4
Professional	76	47.8
Father´s age		
Median, IQR	41	(36–45)
Mother´s education		
Elementary	12	7.6
Graduate	21	13.2
Technical or technological	57	35.9
Professional	69	43.4
Mother´s age		
Median, IQR	39	(33–42)
Marital status		
Married	89	56.0
Cohabiting	26	16.4
Separated	38	23.9
Widowed	6	3.8

**Table 2:** Characteristics of MIH and dental caries

	n	%
MIH		
Healthy	122	76.7
Mild	6	3.8
Severe	31	19.5
Dental caries lesions		
Healthy	144	90.6
Inactive	2	1.3
Active	13	8.2
MIH and dental caries lesions		
No MIH, no dental caries lesions	119	74.8
MIH only		
Mild	6	3.8
Severe	19	11.9
Dental caries lesions only		
Inactive	2	1.3
Active	1	0.6
MIH and caries lesions		
Severe MIH and active lesions	12	7.5

### Outcome

The mean score on the DFA scale was 28.3 (SD = 13.4) with scores ranging from 15 to 64 (Table 3). The mean score on the fear and anxiety scale was higher in females (mean = 30.8) than in males (mean = 26.5), although the difference was not statistically significant.

**Table 3:** Comparison of dental fear and anxiety levels considering the severity of MIH, dental caries, gender and age

	Observed					Adjusted*			
	Median	Diff		CI 95%	*p *value	Diff	CI 95%		*p* value
MIH									
Healthy	25.3	Ref				Ref			
Mild	20.8	− 4.5	− 9.3	0.3	0.069	− 3.8	− 12.4	4.9	0.394
Severe	41.3	16.0	10.9	21.1	< 0.001	23.8	19.3	28.4	< 0.001
Dental caries									
Healthy	27.3	Ref				Ref			
Inactive	21.5	− 5.8	− 15.1	3.5	0.223	− 5.2	− 12.6	2.2	0.169
Active	40.2	12.9	5.9	19.9	< 0.001	18.6	12.0	25.2	< 0.001
MIH and dental caries^+^						28.8	17.5	40.0	< 0.001
dmft									
0	26.5					Ref			
> 0	35.0					7.0	0.8	13.3	0.027
Gender									
Female	30.8					Ref			
Male	26.5					− 2.1	− 5.8	1.5	0.251
Age									
Per additional year						0.5	− 0.8	1.7	0.476

*Adjusted for age, gender, mother’s education and dmft

^+^Interaction term. Evaluated only in the adjusted model

### Main analysis

Table 3 presents the comparison of DFA considering the severity of MIH, dental caries, gender and age. Compared to healthy children, the perception of DFA was significantly higher both in children with severe MIH involvement with a mean adjusted difference of 23.8 points (95% CI 19.3–28.4; *p *value < 0.001) and amongst children with active caries with a mean difference of 18.6 points (95% CI 12.0–25.2; *p *value < 0.001). In children with both MIH and dental caries, the perception of DFA was even higher than amongst children who only had MIH or dental caries. The interaction between these two dental conditions was statistically significant with a mean difference of 28.8 points (95% CI: 17.5–40.0).

The variable of primary dentition caries experience remained statistically significant, with a mean adjusted difference of 7.0 points (95% CI 0.8–13.3; *p *value = 0.027). No statistical and significant associations were observed by gender (*p *value = 0.251) or age (*p *value = 0.476).

The experience of dental caries in the permanent dentition (difference 1.4; 95% CI: -2.0–4.8; *p *value = 0.429) and dental hypersensitivity (difference 0.3; 95% CI: -1.1–1.8; *p *value = 0.640) did not show a statistically significant association with the perception of DFA.

### Other analyses

In the adjusted model, an interaction between MIH and dental caries was identified regarding the perception of DFA. When both conditions were present, the adjusted mean difference was 28.8 points (95% CI: 17.5–40.0; *p *value < 0.001).

## Discussion

In this study, we assessed the perception of DFA in children and found that the highest scores were presented by those with severe MIH, active dental caries and experience of dental caries in the primary dentition. Understanding why children may experience DFA and what the impacts are on their thoughts, feelings, and behaviours is crucial for practicing empathetic and patient-centred clinical care.

The study of DFA in patients with MIH is relevant and necessary, especially after the systematic review and meta-analysis conducted by Reis et al. (Reis et al. 2023). In their study, the authors pointed out serious deficiencies that could compromise the observed results, such as not using specific indices to classify MIH, not considering the severity of MIH or dental caries experience as confounding factors, and using unrepresentative samples (Reis et al. 2023). This strongly impacted the certainty of the evidence, which was classified as low. This means that, so far, the true effect of MIH on DFA in children is unknown. In our study, we attempted to overcome these limitations using specific and validated indices to classify MIH and dental caries lesions, as well as an adequate sample size. These results may help future systematic review and meta-analysis studies obtain more consistent results.

The CFSS-DS was the instrument we used to assess DFA in children. This instrument has appropriate psychometric properties, high reliability and good criterion validity (Aartman et al. 1998; Krikken et al. 2013). In addition to being easy to use, it has been the most widely used scale in the study of DFA in patients with MIH, thus allowing for comparability of results (Reis et al. 2023; Jälevik et al. 2022). Specific and validated indices were used for the assessment of MIH and dental caries lesions by a calibrated examiner, ensuring the reliability and internal validity of our results. Assessing dental hypersensitivity in children affected by MIH is not an easy task (Raposo et al. 2019). In this study, we used a practical and physiologically compatible method with the stimuli routinely received by the dentin-pulp complex and has been frequently reported in clinical studies (Bekes et al. 2017; Raposo et al. 2019; de Farias et al. 2021).

The mean DFA score found in our study was 28.3 ± 13.4, a value that is similar to what has been reported by other authors (Laureano et al. 2020). In our study, we did not observe gender-based differences in DFA levels; however, scientific literature suggests that DFA levels tend to be higher in women than in men (Klingberg and Broberg. 2007). We believe that the perception of DFA can vary amongst individuals and be influenced by sociocultural factors, so it is not necessarily determined by gender. Regarding age, we also did not find differences in DFA levels. The development of fears and anxieties is a part of the normal development of individuals, and scientific research has not yet elucidated a possible relationship between DFA and age (Klingberg and Broberg. 2007; Campbell 2017). In our study, the mother’s level of education influenced children’s perception of DFA. Mothers with a higher level of education have more knowledge about the importance of oral health and, consequently, may have better access to quality dental resources and care. This could ensure that children receive timely and adequate treatment, which could prevent dental problems and reduce the need for painful procedures. In such cases, there may also be more opportunities for open and honest communication, which can help reduce DFA by providing clear and realistic information.

In this study, the highest levels of DFA were found in children with severe MIH and active dental caries lesions, and a history of dental caries in the primary dentition. These results are consistent with those reported by Özükoç, who found significantly higher mean DFA values in patients with severe MIH compared to patients with mild or moderate MIH (Özükoç 2019). On the other hand, Laureano et al. did not find an association between DFA and MIH, possibly because only 19 of 466 patients had severe MIH (Laureano et al. 2020).

In our study, the majority of children with MIH presented severe defects. In these cases, there is a higher probability of finding active dental caries lesions compared to mild defects (Quintero et al. 2022). Thus, the activity of dental caries lesions can significantly impact the severity of enamel hypomineralisations, and according to our results, DFA levels. The inclusion of dental caries as a covariate may elucidate the variance from other studies and the absence of association between MIH and DFA. Children with severe MIH and active caries lesions often experience painful symptoms that are difficult to control due to pulp inflammation and limited action of local anaesthetics. They are also patients who need more frequent operative treatments and retreatments compared to healthy patients. Consequently, the perception of DFA in patients with MIH and active dental caries lesions can be higher and, as already reported in other studies, negatively impacts oral health-related quality of life (Bussaneli et al. 2022; Dantas-Neta et al. 2016). We believe that our results may be influenced by the timing of sample collection, which coincided with the COVID-19 pandemic. During this time, parents avoided or delayed dental care for their children (Campagnaro et al., 2020), leading to exacerbation of certain conditions such as MIH and dental caries lesions.

The relationship between DFA and dental caries has been a controversial issue in scientific literature. According to Kruger et al., dental anxiety is likely a significant predictor of dental caries and may be a risk factor for the incidence of dental caries (Kruger et al. 1998). On the other hand, Abanto et al. did not find an association between dental anxiety and the severity/extent of dental caries (Abanto et al. 2017). Torriani et al. found that in children, factors such as severe dental caries, not visiting the dentist regularly, and experiencing dental pain are associated with a higher prevalence of dental fear (Torriani et al. 2014). It is essential to highlight that children with DFA often avoid dental treatment, and over time, active lesions can progress and cause pain and infection, making exodontia the treatment of choice (Campbell 2017). This partly explains why children with DFA have a high dmft index. From a clinical perspective, the results of our study suggest that patients with severe MIH and active dental caries may require the attention of a professional with specialised training in identifying and managing DFA to provide comfortable, calm and safe dental care.

This study has some limitations to consider in interpreting its results. First, the coronavirus pandemic disrupted patient recruitment, and it is possible that it influenced children’s perception of DFA when returning to dental appointments after the relaxation of restrictions for in-person dental care (De Haro et al. 2022). Second, we did not consider past dental experiences as a confounding variable, and this could strongly influence children’s perception of DFA. Furthermore, whilst this study provides robust evidence regarding associations by making a rigorous classification of MIH, dental caries and measuring DFA with a validated instrument, it is essential that future studies explore other psychological and social characteristics of the child’s and parents’ environment as possible explanations for DFA.

## Conclusion

Considering the limitations of the present study, it has been shown that children with MIH exhibit higher levels of DFA than children without MIH. In addition, conditions associated with MIH, such as the dental caries experience in the primary dentition and the activity of caries lesions, significantly influenced the perception of DFA in children with MIH.

## Data Availability

The data are not publicly available due to privacy and ethical restrictions.

## Declarations

### Permission to reproduce material from other sources

Does not apply.
